# RETRACTION: Analysis of the Association Between Serum Levels of 25(OH)D, Retinol Binding Protein, and Cyclooxygenase‐2 and the Disease Severity in Patients with Diabetic Foot Ulcers

**DOI:** 10.1111/iwj.14947

**Published:** 2024-06-14

## Abstract

**Retraction:** X. Chen, Y. Xu, X. Meng, R. Geng, X. Wang, G. Zhang, and L. Bai, “Analysis of the Association Between Serum Levels of 25(OH)D, Retinol Binding Protein, and Cyclooxygenase‐2 and the Disease Severity in Patients with Diabetic Foot Ulcers,” *International Wound Journal* 21, no. 3 (2023): e14502, https://doi.org/10.1111/iwj.14502.

The above article, published online on 16 November 2023, in Wiley Online Library (http://onlinelibrary.wiley.com/), has been retracted by agreement between the journal Editor in Chief, Professor Keith Harding; and John Wiley & Sons, Ltd. Following an investigation by the publisher, both parties concluded that the peer review process of this article was manipulated. In addition, the authors reported to the journal that values for statistics were not labelled properly in Figures 1, 2, and 3 and that they had misreported data from a cited article. The retraction has been agreed to because the findings reported in the article are not considered reliable. The authors did not respond to the notice of retraction.

**Retraction:** X. Chen, Y. Xu, X. Meng, R. Geng, X. Wang, G. Zhang, and L. Bai, “Analysis of the Association Between Serum Levels of 25(OH)D, Retinol Binding Protein, and Cyclooxygenase‐2 and the Disease Severity in Patients with Diabetic Foot Ulcers,” *International Wound Journal* 21, no. 3 (2023): e14502, https://doi.org/10.1111/iwj.14502.

The above article, published online on 16 November 2023, in Wiley Online Library (http://onlinelibrary.wiley.com/), has been retracted by agreement between the journal Editor in Chief, Professor Keith Harding; and John Wiley & Sons, Ltd. Following an investigation by the publisher, both parties concluded that the peer review process of this article was manipulated. In addition, the authors reported to the journal that values for statistics were not labelled properly in Figures 1, 2, and 3 and that they had misreported data from a cited article. The retraction has been agreed to because the findings reported in the article are not considered reliable. The authors did not respond to the notice of retraction.

